# The Hydric Environment: A Hub for Clinically Relevant Carbapenemase Encoding Genes

**DOI:** 10.3390/antibiotics9100699

**Published:** 2020-10-15

**Authors:** Florence Hammer-Dedet, Estelle Jumas-Bilak, Patricia Licznar-Fajardo

**Affiliations:** 1 UMR 5569 HydroSciences Montpellier, Université de Montpellier, CNRS, IRD, 34090 Montpellier, France; florence.hammer-dedet@umontpellier.fr (F.H.-D.); estelle.bilak@umontpellier.fr (E.J.-B.); 2 Département d’Hygiène Hospitalière, CHU Montpellier, 34090 Montpellier, France

**Keywords:** carbapenemase, aquatic environment, one-health, horizontal gene transfer

## Abstract

Carbapenems are β-lactams antimicrobials presenting a broad activity spectrum and are considered as last-resort antibiotic. Since the 2000s, carbapenemase producing *Enterobacterales* (CPE) have emerged and are been quickly globally spreading. The global dissemination of carbapenemase encoding genes (CEG) within clinical relevant bacteria is attributed in part to its location onto mobile genetic elements. During the last decade, carbapenemase producing bacteria have been isolated from non-human sources including the aquatic environment. Aquatic ecosystems are particularly impacted by anthropic activities, which conduce to a bidirectional exchange between aquatic environments and human beings and therefore the aquatic environment may constitute a hub for CPE and CEG. More recently, the isolation of autochtonous aquatic bacteria carrying acquired CEG have been reported and suggest that CEG exchange by horizontal gene transfer occurred between allochtonous and autochtonous bacteria. Hence, aquatic environment plays a central role in persistence, dissemination and emergence of CEG both within environmental ecosystem and human beings, and deserves to be studied with particular attention.

## 1. The Carbapenem Resistance Issue: The Importance to Mesh the Aquatic Environment

Antimicrobials are obviously one of the most significant discovery of the past century. Their use has allowed important advances in preventing or delaying the onset of bacterial infections in medicine and veterinary practices, and have also been largely used as prophylactic and growth promoter agents in animal husbandry and farming. However sometimes, acquisition of antimicrobial resistance (AMR) determinants by bacteria occurs, either by selection of resistant bacterial mutant or by horizontal transfer of AMR determinant-encoding genes within bacterial species [1]. Due to the overuse and the misuse of antimicrobials, a selective pressure places to bacteria enhancing dramatically AMR that results in failures of antimicrobial treatment efficacy. By far, β-lactams are the most consumed antibiotic worldwide [2]. This class of antibiotic includes penicillins, cephalosporins, monobactams and carbapenems. Carbapenems, such as imipenem or meropenem, present the broadest spectrum of activity and a bactericidal activity against many Gram-negative and Gram-positive bacteria [3,4]. Their particular chemical structure confers protection against most of the β-lactamases produced by bacteria including cephalosporinases or extended-spectrum β-lactamases (ESBL) [4,5]. Consequently, carbapenems are considered as last-resort antibiotics and are used to treat bacterial infections when all other therapeutic options fail [5]. Therefore, bacterial carbapenem resistance is an important public health concern. Among carbapenem-resistance determinants, carbapenemase enzymes are the most relevant clinically and the most worrying because; (i) these enzymes hydrolyse almost all β-lactams, including carbapenems that limit severely therapeutics options; and (ii) clinically relevant carbapenemases are encoded by genes located onto genetic mobile elements, such as transposons or plasmids and are horizontally transferable within different bacterial species. This is particularly true for Gram-negative bacteria, especially for Carbapenemases Producing *Enterobacterales* (CPE) that have emerged in the 2000s, have quickly globally spread, and are associated with health-care associated infection and important mortality rate [6,7].

In the last decade, clinically relevant CPE have been isolated from non-human sources, including animals, food chain and the environment [8], highlighting the necessity to consider human, animal and environmental interfaces for the struggle against AMR under the holistic approach named the «One-Health» concept [1,9]. Among components of the «One-Health» triad, the environment and more particularly the aquatic environment represents a crucial component as it is strongly linked with anthropic activities. These activities lead to multiple selection pressures exerted on the bacterial community [1,10], as well as meetings and exchanges between human and environmental bacteria. Urban aquatic environments are particularly impacted by anthropic activities, such as the discharge of habitations, urban runoffs, and hospital sewages [11,12,13,14,15], wastewater treatment plants (WWTP) effluents [13,16,17] and domestic or recreational activities [13,18]. While, urbanization of human habitats is on the rise, rural hydric environments are also impacted by animal husbandry and farming activities [19]. Then, humans could be exposed to Carbapenemase Producing Bacteria (CPB) including CPE, contained in aquatic environment, either directly during recreational activities, after flood episodes, or simply by drinking tap water [20,21,22], and either indirectly by foodborne transmission (re-use of treated wastewater for agricultural and livestock activities) [23,24]. Therefore, mixes between aquatic environmental communities and bacterial communities from human beings are consequently bidirectional and aquatic ecosystems constitute a hub for CPB. Moreover, these aquatic environments could act as reservoir for clinically relevant CPB, but also for Carbapenemase Encoding Genes (CEG). Since it contains diverse autochtonous bacteria which could exchange genetic material by Horizontal Gene Transfer (HGT) with allochtonous bacteria (e.g., CPE from human or animal microbiota) and vice versa, aquatic environment plays a central role in persistence, dissemination and emergence of CEG both, within environmental ecosystem and human beings and deserves to be studied with particular attention.

This paper therefore seeks to provide justification for considering potential roles of aquatic autochtonous bacteria in the emergence, the dissemination and the persistence of CEG and the requirement to study CPB in aquatic ecosystem for the survey and the prevention of AMR. 

## 2. Carbapenemases: A Clinical Success Story

Carbapenems are hydrolysed by some specific β-lactamases called carbapenemases. β-lactamases are shelved by different classifications. The Ambler classification [25], based on the primary structure of the enzymes, is the most popular and describes four classes of β-lactamases. Only three of them, A, B and D, contain clinically relevant carbapenemases and are described below. Class C enzymes are not considered as carbapenemases as they harbour a low hydrolysis potential of carbapenems [26].

### 2.1. Class A

Class A carbapenemases are serine proteases which hydrolyse with greater or lesser degree penicillins, monobactams, cephalosporins and carbapenems and are partially inhibited by β-lactamase inhibitors (e.g., clavulanic acid, sulbactam, tazobactam) [5]. Some of enzymes of this class are chromosomally encoded, not transferable and confer an inherent resistance to carbapenems to bacteria species that carry them. As example, one can list SME (*Serratia marcescens* enzyme) and SFC-1 (*Serratia fonticola* carbapenemase A) for *Serratia* spp., or NmcA (not metalloenzyme carbapenemase A) or IMI-1/IMI-2 (imipenem-hydrolysing β-lactamase) for *Enterobacter* spp. Other class A carbapenemases are plasmid-encoded like KPC (*Klebsiella pneumoniae* carbapenemase), IMI (IMI-1 to IMI-3) or derivatives of GES (Guiana extended spectrum) [5].

Among these class A-enzymes, KPC is the most worrisome because of its current widespread within clinical *Enterobacterales* [5,27,28]. KPC enzyme was described for the first time in a *Klebsiella pneumoniae* isolate in United States in 1996. This discovery was quickly followed in 2001 by the description of a novel single amino-acid variant KPC-2 [29] which became global in less than ten years [30]. It is worth noting that recent resequencing of *bla*_KPC-1_ revealed that it was identical to *bla*_KPC-2_ and thus KPC-1 and KPC-2 variants are identical too [31]. Since then, more than 44 KPC variants have been described but do not harbour the same ability to hydrolyse carbapenems. For instance, some of them such as KPC-31, KPC-33, KPC-14 or KPC-28 are not considered as carbapenemase enzymes [30] while KPC-2 and KPC-3 present a broad spectrum of hydrolysis of β-lactams and are from the most frequent variants encountered in clinically strains [32].

**Figure 1:**
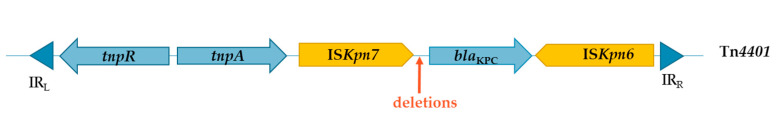
Diagram of Tn*4401* structure, according to Nordmann et al., 2011 [32] and Partridge et al., 2018 [36]. Genes and their corresponding transcription orientation are indicated by large blue arrows. The Tn*4401* is delimited by two inverted repeat (IR) sequences, represented by dark blue triangles. Tn*4401* carries *bla*_KPC_ gene, it is a Tn3-type transposon which possess a resolvase and a transposase respectively encoded by *tnpR* and *tnpA* and two insertion sequences (yellow large arrows) IS*Kpn6* and IS*Kpn7*. Approximative position of deletions leading to different variants of Tn*4401* are represented by the thin orange arrow.

The global success of the KPC-2 spread within clinical *Enterobacterales* could be owed to; (i) its frequent association with the nosocomial successful *K. pneumoniae* sequence type (ST) 258 [33,34,35]; and (ii) the particular genetic context of its encoding gene (Figure 1). Indeed, *bla*_KPC-2_ is located on Tn*4401*, a Tn*3*-type transposon which possesses a resolvase (*tnpR*), a transposase (*tnpA*) and two insertion sequences IS*Kpn6* and IS*Kpn7.* Different variants of Tn*4401* carrying different internal deletions have been identified. A nomenclature with lowercase letter is employed to distinguish these variants. The most common form is Tn*4401a* which gives the highest level of carbapenem resistance [36]. Tn*4401* exhibits a high transposition frequency and allows the dissemination of *bla*_KPC-2_ gene within a large range of plasmids and bacterial species [37,38]. Therefore, it is also encountered in bacteria isolated from hydric environments.

For instance, Mathys and colleagues have isolated different bacterial species which carry *bla*_KPC_ genes on different classes of plasmids from WWTP effluents [23]. In their study, Sekizuka et al. identified similar IncP-6 plasmids carrying *bla*_KPC-2_ genes in *Klebsiella* sp., *Citrobacter* sp., *Enterobacter* sp., *Escherichia coli*, *Pseudomonas* sp., *Aeromonas caviae* and *Aeromonas hydrophila* from WWTP effluent [39]. A Chinese study identified an IncP-6 plasmid carrying *bla*_KPC-2_ gene in three isolates of *Aeromonas taiwanensis* isolated from sediments of an urban river. Interestingly the *bla*_KPC-2_ gene was inserted in a Tn*3* transposon as previously reported in clinical *Enterobacterales* in China and in France [40]. *bla*_KPC_ genes have also be found to be carried by a conjugative plasmid in a *K. pneumoniae* strain isolated from urban lake water [41] and in *Citrobacter freundii* and *A. caviae* strains isolated from an urban river close to a WWTP [42].

### 2.2. Class B

Carbapenemases belonging to Ambler’s class B are metallo-enzymes (also called “metallo-β-lactamases” MBL). Its active site includes at least one zinc ion Zn(+II). Most of these enzymes present a broad lysis spectrum for β-lactams. Their activity is not inhibited by the commercially available β-lactamases inhibitors but they are susceptible to ethylenediaminetetraacetic acid (EDTA), a chelator of divalent cations [5,43].

Until the early 1990s, MBLs described and studied were exclusively chromosomally encoded, conferring a species-specific resistance to carbapenems. These enzymes are widespread in environmental bacterial species such as L1 and L2 enzymes in *Stenotrophomonas maltophilia*, CphA in *Aeromonas* spp. (*A. hydrophila*, *Aeromonas sobria*, *Aeromonas salmonicida*) or β-lactamase II in *Bacillus cereus* [44,45,46,47]. Since then, a wide variety of genetic mobile element structures carrying MBL-encoding genes were identified. The most common embrace Verona Integron-encoded Metallo-β-lactamase (VIM), Imipenem-resistant *Pseudomonas* (IMP), Seoul Imipenemase (SIM), German Imipenemase (GIM) and the successful New Delhi Metallo-β-lactamase 1 (NDM-1).

The NDM-1 enzyme is an MBL which presents a broad lysis spectrum, hydrolysing all β-lactams except aztreonam. It was identified for the first time in 2009 in India, in a carbapenem-resistant *K. pneumoniae.* This strain was involved in a urinary tract infection of a Swedish patient travelled in New Delhi. The *bla*_NDM-1_ gene has also been identified in an *E. coli* strain isolated from faeces of this patient. These *bla*_NDM-1_ genes were on different transferable plasmids [48]. Since then, spreading of *bla*_NDM-1_ gene has occurred within clinically relevant bacteria in particular in *Enterobacterales*, *Acinetobacter* spp. and *Pseudomonas* spp. [49,50,51,52] and until now 29 variants of NDM-1, varying by few amino-acid residues at different positions have been described [53]. Several plasmids types harbouring *bla*_NDM-1_ gene have been identified suggesting that plasmids play a major role in the rapid dissemination of this gene to the global scale [49,50,51,52,53,54,55]. Among *bla*_NDM-1_ carrying-plasmids, IncA/C plasmid type, a Broad Host Range (BHR) plasmid, generally conjugative, is frequently described [51,53,56,57]. This argues the involvement of this plasmid in *bla*_NDM-1_-dissemination among different bacterial species. Some narrow-host-range plasmid are also involved in NDM dissemination. For instance, IncX3-type plasmid that is restricted to *Enterobacterales*, has been found in association with *bla*_NDM-5_ gene in several clinical isolates in China [57,58], including ones with the same genetic environment that an IncX3-type plasmid circulating in India and in Japan [58]. This suggests that IncX3-type plasmids may play a role in the spread of *bla*_NDM_ (at least *bla*_NDM-5_) genes within *Enterobacterales*. Less frequently, chromosomic *bla*_NDM-1_ gene was identified, associated with different transposons particularly in *Pseudomonas* spp. and *Acinetobacter* spp. [59,60]. In addition of plasmids and transposons, insertion sequences (IS) may have participated to the spread of *bla*_NDM-1_ gene too. The association of *bla*_NDM-1_ with at least a remnant of IS*Aba125* in several bacterial species in particular *Enterobacterales* and *Acinetobacter* spp. has been noted [53,54,60,61]. In contrast, even if integrons are mobile genetic elements massively involved in AMR dissemination, integrons do not seem to be linked with *bla*_NDM_ dissemination. A study evaluating the genetic context of clinical NDM-producing *Enterobacterales* strains has shown no relation between *bla*_NDM_ gene and class 1 integron occurrences [54]. Therefore, NDM-1 encoding gene is not associated with a particular plasmid type or genetic mobile element and the genetic context of *bla*_NDM-1_ gene is inconstant [62].

While, clinical bacteria producing NDM enzyme are broadly described, only few studies reported the isolation of NDM producers in aquatic environment. *bla*_NDM_ genes have already been identified in *Aeromonas* spp., *Acinetobacter* spp. and *Citrobacter* spp. from hospital sewage effluent [11,12] and different *Enterobacterales* species producing NDM-1, NDM-5 and NDM-7 enzymes were isolated from WWTP effluent [23]. Recently, two isolates of *E. coli* carrying *bla*_NDM-5_ gene onto IncX3-type plasmids were isolated from an urban river in the South of France [63].

### 2.3. Class D

Class D β-lactamases are serine protease enzymes which hydrolyse preferentially oxacillin rather than benzylpenicillin, hence their usual name is OXA enzymes [64]. These enzymes are generally poorly inhibited by EDTA and penicillinase inhibitors (i.e., clavulanic acid, sulbactam, tazobactam), but their action is suppressed by avibactam [43,65]. As amino-acid sequences of these enzymes are variable, hydrolysis spectrum is also variable and some of them are able to hydrolyse carbapenems. They are named carbapenem-hydrolysing class D β-lactamases (CHDB). CHDB enzymes were identified primary from non-fermenter organisms, such as *Acinetobacter baumannii*: OXA-24, OXA-25, OXA-26, OXA-27, OXA-40 and OXA-23 (firstly named ARI for *Acinetobacter* Resistant Imipenem) [64,66]. Ten years after the identification of the first CHDB [67], a novel one, the OXA-48 exhibiting a high level of resistance to all β-lactams, including carbapenems was identified from a multidrug-resistant *K. pneumoniae* isolate responsible of an urinary tract infection in a patient hospitalized in Istanbul [66]. The OXA-48 enzyme was weakly related to other oxacillinases, including CHDB, with a maximum of 46% of amino-acid identity. It has been shown that OXA-48 hydrolyses carbapenems at a higher level of efficiency than others CHDB previously described. Hydrolysis efficiency of OXA-48 was comparable to that of KPC enzymes [66]. Like other CHBD, OXA-48’s activity is not affected by EDTA and by penicillinase inhibitors but is characterized by a sensitivity to NaCl conferred by the mutation Y144F [66,68]. Since then, several variants of OXA-48 enzymes, also named OXA-48 like enzymes, have been described in *Enterobacterales* isolates, OXA-181, OXA-232, OXA-244, OXA-204 and OXA-162 being the most common ones [69]. Unlike most oxacillinase-encoding genes described previously, the *bla*_OXA-48_ gene was not part of a class 1 integron but was a plasmid-borne gene [66]. For a long time, *bla*_OXA-48_ gene (and its variants) have been identified only in *Enterobacterales* [70] and its dissemination was exclusively associated with a single pOXA-48a plasmid. Complete sequencing of pOXA-48a plasmid highlighted that it was a 62.3 kb IncL/M-type plasmid and *bla*_OXA-48_ was located within the transposon Tn*1999* [71]. IncL/M-type plasmids are BHR plasmids, commonly identified in *Enterobacterales* and associated with a variety of Antimicrobial Resistance Gene (ARG) [72]. Tn*1999* is a composite transposon, formed by two identical IS*1999* [73] and the *bla*_OXA-48_ gene. *bla*_OXA-48_ gene is located downstream one of the IS*1999* which provides a promoter sequence responsible of its expression [66]. Since then, lots of studies have reported OXA-48-producing *Enterobacterales* (mostly *Klebsiella* sp.) in many countries. OXA-48 is actually the most prevalent carbapenemase in Middle East, North of Africa and Europe [69,70]. The pOXA-48 like plasmids are still considered the main source of *bla*_OXA-48_ dissemination within clinical *Enterobacterales* [74] and in different ecological niches including urban hydric environments [8,75,76]. Interestingly, some studies reported the occurrence of non-fermenter bacteria that carry *bla*_OXA-48_ gene such as *Acinetobacter* sp. and *Pseudomonas* sp. from potable tap water in US or *Aeromonas* sp. and *S. maltophilia* from biofilms of hospital effluent. Unfortunately, the genetic background of these genes have not been studied yet [11,77].

## 3. Presence of CPB in Aquatic Environments: A Simply Remnant of Human Activities or an History a Little Bit More Complex Than That?

### 3.1. Emergence of CPE in Aquatic Environments: As a Consequence of Anthropic Activities

A number of studies reported the isolation of CPE from urban aquatic environments such as surface water nearby WWTP [23], rivers [13,42,63,78,79,80,81,82,83], lakes [15,18,41] or coastal water [15,18]. In most of cases, the origin of CPE is recognized or strongly suggested to come from anthropic contaminations. The isolation of CPE such as *Enterobacter* sp. [78], *Klebsiella* sp. [13] and *E. coli* [63] carrying acquired CEG has been noted from urban river water considered to be impacted by anthropic activities.

Discharging habitation sewage and WWTP effluent are probably two of the main reasons for the presence of CPE in urban rivers. A study reports the isolation of *Klebsiella* spp. carrying *bla*_KPC-2_ gene from river water samples and also shows a deterioration of the water quality along the river downstream of the dwellings [13]. Previous studies have reported the occurrence of CPE in WWTP effluent and in aquatic environment nearby WWTP (or in which WWTP effluent are discharged) [17,23,42]. Hospital sewage wastewaters are also a source of contamination for urban aquatic environment. Hospital sewage is considered a hotspot for HGT of ARG since its contains multi-resistant bacteria, viruses [84], antibiotics and biocides [14]. CPE is also commonly present in hospital sewage wastewater [85]. As an example, *K. pneumoniae* belonging to high-risk hospital associated lineage (CC11, CC258) carrying plasmidic *bla*_KPC-2_ gene has been reported in urban aquatic areas close to hospital settings [41,82].

While, the most likely reason for the occurrence of CPE in urban water is an anthropic contamination, it is also possible that aquatic environment contains persistently CPE, which can act as a reservoir and could play the role of a source for acquisition of CEG by other bacteria through HGT, and for future human colonization.

### 3.2. Providing Persistence and Dissemination of CEG in Aquatic Environments: As an Opportunity for CPE

In a significant way, some of *Enterobacterales* commonly associated with aquatic environment could constitute a reservoir for CEG in water. This is the case for species belonging to the genus *Enterobacter*, which are commonly found in the environment and are associated with various habitats, such as water, soil, animals and plants [86,87]. *Enterobacter* species are occasionally pathogens for human and are involved in nosocomial infection (especially in immunocompromised patients). Several studies have reported clinical isolates carrying carbapenemase encoding genes like *bla*_IMI_, *bla*_NDM_, *bla*_OXA-48_, *bla*_VIM_ or *bla*_KPC_ [86,87,88,89]. Beside these clinical reports, the occurrence of carbapenemase-producing *Enterobacter* spp. has been notified in urban aquatic environment such as WWTP effluent, surface water, coastal water, hospital effluent and rivers [12,13,15,16,18,23,78,88,90,91]. Interestingly, some studies emphasize the persistence of *Enterobacter* spp. carbapenemase-encoding strains in rivers which allows to evoke its potential role as reservoir for CEG in aquatic ecosystems. Aubron et al. mentioned the presence of *Enterobacter cloacae* isolates clonally related and carrying *bla*_IMI-2_ gene on a plasmid from four distantly related rivers in United States [78]. The persistence of a clonal complex of KPC-2 carbapenemase-producing *Enterobacter asburiae* along an urban Brazilian river was also reported [13].

Persistence of CPE in aquatic environment and their potential transmission to human is worrying and the case of an infection after a near-drowning caused by a persistent strain of IMI-2 carbapenemase-producing *E. asburiae* in a river of the South of France has already been reported [88]. 

Other *Enterobacterales* could play the role of environmental reservoir of resistance genes, particularly when presenting an aquatic habitat and when some strains isolated from water are known to carry carbapenemase encoding genes [12,15,16,18,23,42,92]. It is quite likely that CPE, regardless their origin, may play a crucial role in the persistence and the dissemination of CEG through aquatic environment to the human being. Limiting these roles to only CPE is probably an error since several studies reported the occurrence of carbapenemase-producing autochtonous bacteria not belonging to *Enterobacterales* order in diverse aquatic environments [11,12,13,15,16,18,23,39,42,77,91,93,94,95]. This should give us the opportunity to consider potential roles played by aquatic autochtonous CPB in the emergence, the dissemination and the persistence of CEG.

### 3.3. Carbapenemase-Producing Environmental Bacteria: The Chicken or the Egg?

Well before their discovery and their use by humans, antimicrobials were already naturally synthesized in the Environment. In fact, microorganisms are in competition for the access to nutrient and oxygen and many of them produce molecules, in order to control competitor development. For example, thienamycin (i.e., the first carbapenem molecule identified) is constitutively produced by *Streptomyces cattleya*, a soil environmental bacteria [96]. To protect themselves against molecules that they produce, microorganisms synthesize resistance determinants, encoded by genes generally located onto the chromosome and transmitted to progeny (i.e., vertical transmission). However, microorganisms which not produce antimicrobials can also present constitutively ARG and/or acquire de novo new ones by HGT. Therefore, it might be speculated that some CEG (like others ARG) have been mobilized from environmental bacteria and that, in a manner of speaking, environmental autochtonous bacteria could be «the egg» at the origin of CEG. Supporting this idea, it is well accepted in the scientific community that *Shewanella* sp., a waterborne bacterial genus, is at the origin of *bla*_OXA-48_ like encoding genes [76,95,97]. More putatively, some authors suggest that *A. baumannii* may constitute an intermediate reservoir for *bla*_NDM-1_ gene [49,60,98]. By the analysis of *bla*_NDM-1_ sequence, Bonnin et al. imply that *bla*_NDM-1_ come originally from an environmental progenitor bacteria and has been constructed in *Acinetobacter* by a recombination event with another resistance gene. Moreover, in *A. baumannii*, *bla*_NDM-1_ gene is embedded in a transposon composite Tn*125*, which may have then allowed the transfer of the gene onto BHR plasmids and its secondary dissemination by HGT within *Enterobacterales* [60].

Autochtonous bacteria can also acquire new CEG. Environmental aquatic bacterial genera, which acquired clinical relevant CEG from contaminated water have been reported in many previous studies [11,13,15,16,39,40,94,99]. As autochtonous bacteria are adapted to persist in the aquatic environment, they can constitute a perennial environmental reservoir for CEG. However, the direction of the CEG transfer between human being and aquatic environment is probably not unidirectional. It could also occurs from autochtonous bacteria to human bacteria. This must then drive us to consider autochtonous bacteria like a potential “shuttle” for CEG which could ensure the transfer of the genes within aquatic and human environments.

## 4. The Bacterial Shuttle Concept: When Hydric Autochtonous Bacteria Play a Role of “Genes-Mule”

Some aquatic autochtonous bacteria are opportunistic pathogens that can cause human infection or colonization, and can transfer CEG by HGT to bacteria belonging to host microbiota (i.e., humans). These aquatic autochtonous bacteria could act as reservoirs, and more particularly, like a hub or a “bacterial shuttle” for CEG, permitting them to travel from hydric environment to the human being (and vice versa) and to persist and disseminate within humans. This is particularly true if the resistance genes transferred are clinically relevant and are located onto mobile genetic elements which facilitate their dissemination between human bacteria.

As CEG are generally carried onto transposable elements and plasmids and as HGT occurs frequently between different bacterial species belonging to *Enterobacterales* [28,100], the example of aquatic autochtonous CPE as “bacterial shuttle” appears to be the most obvious. Supporting this idea, Potron et al. suggest a wide dissemination of an OXA-48 producing-*Serratia marcescens* strain in urban puddles in Marrakech. The *bla*_OXA-48_ gene was encoded on a conjugative plasmid and was embedded in the same Tn*1999* transposon that has already been isolated in *K. pneumoniae* strains in the country [92]. The description of a *bla*_IMI-2_ gene flanked by transposable elements on a conjugative plasmid in a strain of *E. asburiae* [89] supports also this hypothesis for this bacterial species. However we chose here to focus on two bacterial genera not belonging to *Enterobacterales*: *Aeromonas* and *Pseudomonas*. In fact, *Aeromonas* and *Pseudomonas* are considered to be opportunistic pathogens for humans and are frequently associated with aquatic habitat and thus should be considered as good candidate to be “bacterial shuttles” and deserve special attention.

### 4.1. Aeromonas *Spp.*

*Aeromonads* belong to *Gammaproteobacteria*. These non-fermentative Gram-negative rods are ubiquitous and go hand-in-hand with water and aquatic environments: Frequently isolated in fresh water like rivers and lakes, drinking water, wastewater, sewage and more rarely in seawater [101]. *Aeromonas* spp. are opportunistic pathogens and can be associated with gastroenteritis, blood-born infections, skin and soft tissue infections. The main source for human colonization/infection is contact with contaminated water directly (e.g., recreational water, drinking water) or indirectly (e.g., animal, foodborne…) [101]. Some species of *Aeromonas* sp., including *A. hydrophila* are naturally resistant to carbapenems, due to the chromosomally mediated gene *cphA* encoding an Ambler class B carbapenemase [47].

Some studies report isolation of *Aeromonas* spp. strains carrying acquired carbapenemase encoding gene from urban aquatic environment, such as WWTP effluent, river or coastal water [12,13,15,16,39]. In a Japanese study, *A. hydrophila* and *A. caviae* carrying *bla*_KPC-2_ gene on different IncP-6 plasmids were isolated from WWTP effluent and the authors suggested that the acquisition of the carbapenemase encoding gene occurred in the aquatic environment and *Aeromonas* may constitute an environmental reservoir for KPC-2 carbapenemase encoding genes [39]. Even if there is no clear proof that the gene transfer from clinical bacteria to *Aeromonas* spp. (naturally present in the aquatic environment) took place in the water, other studies also suggest it. A Chinese study identified of an IncP-6 plasmid encoding *bla*_KPC-2_ gene in three *A. taiwanensis* strains isolated from sediments of an urban water. Interestingly, the *bla*_KPC-2_ gene was inserted in a transposon Tn*3*, which had already been reported in *Enterobacterales* in China and in France [40]. Others studies noticed environmental *Aeromonas* spp. isolates carrying *bla*_KPC-2_ gene on self-transmissible plasmids in urban water [16,42]. In the same way, Mathys et al. isolated 31 *Aeromonas* spp. strains producing carbapenemase from WWTP effluent, 5 carrying *bla*_KPC-2_, 4 onto an IncP-6 plasmid [23]. A French study reported the isolation of several *Aeromonas* spp. strains carrying plasmidic carbapenemase encoding genes (*bla*_NDM_, *bla*_OXA-48_, *bla*_VIM_) from old biofilms formed on hospital wastewater pipelines. One of these plasmids harbouring *bla*_VIM-19_ gene, was a conjugative plasmid [11]. In their study, Picão et al. isolated several CPB from different stages of water purification. The genetic environment of all the *bla*_KPC_ carriers were characterized. Two different genetic environments were evidenced, a Tn*4401*, mostly carried by *Enterobacterales* commonly implicated in human pathology (*Klebsiella* spp., *Raoultella* spp., *Enterobacter* sp. *Citrobacter* spp.) and a Tn*3*-*tnp*R/IS*Kpn*8/*bla*_KPC-2_/IS*Kpn*6 array in *Aeromonas* spp. Interestingly, *Aeromonas* sp. is the only genus for which the two transposons has been identified [16].

The fact that autochtonous bacteria carry CEG on transferable plasmids already identified in CPE involved in human infection reinforces the idea that HGT between hydric and human bacteria can occur in aquatic environments and in humans after a waterborne infection (“bacterial shuttle” concept). 

Clinical *Aeromonas* spp. isolates producing KPC-2, VIM, OXA-181 and GES-24 carbapenemases have already been identified in US, Israel and Japan [102,103,104,105]. A clinical case of HGT of a *bla*_VIM-1_ gene carried by a conjugative IncA/C plasmid was established in vivo between 3 *Enterobacteriaceae* isolates (*E. coli*, *K. pneumoniae* and *C. freundii*) and an *A. hydrophila* isolate. Even if the route of the HGT between the four isolates could not be determined, this study proves that HGT of carbapenemase encoding gene between an aquatic bacteria (acquired after a water exposition) and more clinically relevant bacteria (probably belonging to the microbiota of the patient) is possible within a patient [106]. An HGT was suggested to occur between a human strain and an environmental strain within the intestinal tract of an hospitalized patient. In detail, this study described the transfer of a plasmid carrying *bla*_TEM-24_ (ESBL encoding gene) from an isolate of *Enterobacter aerogenes* (belonging to a lineage associated with TEM-24 in French hospital) to an isolate of *A. caviae* [107].

### 4.2. Pseudomonas *Spp*.

*Pseudomonas* spp. are aerobic Gram-negative non-fermentative bacilli belonging to *Gammaproteobacteria*. *Pseudomonas* spp. are considered as ubiquitous bacteria and are able to colonize a wide range of habitats like soil, plants, animals and water [108]. Although, *Pseudomonas aeruginosa* is one of the principal agent responsible for hospital acquired infection [109,110], the other species of the genus (hereinafter called *Pseudomonas* sp. *non aeruginosa*) exhibit limited pathogenicity to humans [111]. *Pseudomonas* AMR is mainly studied for *P. aeruginosa*, where acquired resistance to carbapenems could occur i) by mutation onto genes conducting to overexpression of efflux system or to overexpression of the cephalosporinase AmpC or to modification of porin protein D2 and ii) by HGT [110]. Acquisition of CEG by HGT is largely described in *P. aeruginosa* clinical isolates and the expansion of epidemic high-risk clones producing carbapenemase is increasing in the world [92]. Resistance by carbapenemase production in *Pseudomonas non aeruginosa* is less described in clinical reports. However, the carriage of CEG like *bla*_KPC-2_ and *bla*_VIM-2_ (carried on an class 1 integron) respectively in a clinical isolates of *Pseudomonas putida* and *Pseudomonas fulva* has been noticed [111,112].

Interestingly, a substantial number of *Pseudomonas non aeruginosa* isolates carrying acquired CEG were isolated from various aquatic environment. This suggests that the *Pseudomonas* genera may act as an environmental reservoir for the persistence and the spread of CEG in aquatic bacterial community [11,12,15,93,94,99,113,114]. Moreover, it is to note that *Pseudomonas* spp. have a propensity to form biofilm [11,108] which is recognised to be a site where there is numerous exchanges of genetic material in particular by HGT [115].

When the genetic context of the carbapenemase encoding gene is studied, most of the studies report an association with the integron of class 1. In their study, Quinteira et al. described a *Pseudomonas pseudoalcaligenes* carrying a chromosomal encoded gene-*bla*_VIM-2_ in a class 1 integron isolated from an urban sewage system [116]. As *P. pseudoalcaligenes* is an environmental bacteria widely distributed in aquatic habitats but rarely implicated in clinical infection, and because there was no VIM-2 producing-*P. pseudoalcaligenes* clinical strain isolated in the hospital during the study, the authors argued for an HGT occurrence in the aquatic environment and for a distribution of *bla*_VIM-2_ not restricted to hospital settings [116]. Another study reported the isolation of 141 *Pseudomonas* spp. strains from aquatic sediments receiving communal and hospital (treated or untreated) effluents. Among these isolates, the authors noted an important proportion of the carbapenemase encoding genes *bla*_VIM-1_ (20 to 61%) and *bla*_NDM-1_ (6 to 29%) and almost one third carrying a class 1 integron; *bla*_NDM-1_ was recovered in various *Pseudomonas species* including *P. aeruginosa* but more surprisingly also *P. putida*, *P. fulva* and *Pseudomonas plecoglossida*, which are species rarely involved in human pathology. That is why authors suggested a transfer of this gene to aquatic *Pseudomonas* community [117]. The same study reports the success of the transfer by in vitro conjugation to *E. coli* of *bla*_VIM_ and *bla*_NDM_ genes from *P. aeruginosa* and *P. putida* (with a higher transfer rate at 30 and 37 °C). This major result proved that HGT can occur between *Pseudomonas* spp. and *Enterobacterales* and that the potential role of «shuttle» between aquatic environment and human being for bacteria belonging to *Pseudomonas genus* appears plausible.

## 5. Conclusions

There are many reasons justifying the presence of CPB in aquatic environments is a great concern. The first one, and the most obvious, is that contaminated water can provide CPE to humans that can cause bacterial infections or colonization for humans. These CPE could then transfer their CEG to aquatic autochtonous bacteria that provoke the persistence and the dissemination of the CEG within aquatic ecosystem, illustrating the bacterial shuttleconcept. Moreover, aquatic autochtonous bacteria can act also as shuttle for carbapenemase encoding genes between aquatic environment and human being as it has been already suggested [88]. Humans are in contact with water in several ways and the transient implantation in the microbiota of autochtonous bacteria after a near drowning has already been noticed [88,106], which strongly allows a potential transfer of genes from autochtonous bacteria to human bacteria in the gut. 

Most of the available studies chose to focus onto the clinical relevant bacterial species, which produce carbapenemase excluding de facto ones that are unlikely found in human infections [23,41]. But given the fact that, autochtonous bacteria are probably a shuttle, the restricted evaluation of clinical relevant bacteria (most of the time *Enterobacterales*) producing carbapenemase grossly underestimates the risk for human health. In the context of the fight against AMR under the “One-Health” concept, when the emergence, the dissemination and the persistence of CEG and more generally of AMR genes is studied in the Environment, bacterial “shuttle” concept and autochtonous bacteria appear essential to be considered.
